# Automated Evaluation of Conventional Clock-Drawing Test Using Deep Neural Network: Potential as a Mass Screening Tool to Detect Individuals With Cognitive Decline

**DOI:** 10.3389/fneur.2022.896403

**Published:** 2022-05-03

**Authors:** Kenichiro Sato, Yoshiki Niimi, Tatsuo Mano, Atsushi Iwata, Takeshi Iwatsubo

**Affiliations:** ^1^Department of Neuropathology, Graduate School of Medicine, The University of Tokyo, Bunkyo, Japan; ^2^Unit for Early and Exploratory Clinical Development, The University of Tokyo Hospital, Tokyo, Japan; ^3^Department of Neurology, Graduate School of Medicine, The University of Tokyo, Bunkyo, Japan; ^4^Department of Neurology, Tokyo Metropolitan Geriatric Center Hospital, Tokyo, Japan

**Keywords:** deep learning, screening, cognitive decline, dementia, clock drawing test (CDT)

## Abstract

**Introduction:**

The Clock-Drawing Test (CDT) is a simple cognitive tool to examine multiple domains of cognition including executive function. We aimed to build a CDT-based deep neural network (DNN) model using data from a large cohort of older adults, to automatically detect cognitive decline, and explore its potential as a mass screening tool.

**Methods:**

Over 40,000 CDT images were obtained from the National Health and Aging Trends Study (NHATS) database, which collects the annual surveys of nationally representative community-dwelling older adults in the United States. A convolutional neural network was utilized in deep learning architecture to predict the cognitive status of participants based on drawn clock images.

**Results:**

The trained DNN model achieved balanced accuracy of 90.1 ± 0.6% in identifying those with a decline in executive function compared to those without [positive likelihood ratio (PLH) = 16.3 ± 6.8, negative likelihood ratio (NLH) = 0.14 ± 0.03], and 77.2 ± 2.7 % balanced accuracy for identifying those with probable dementia from those without (PLH = 5.1 ± 0.5, NLH = 0.37 ± 0.07).

**Conclusions:**

This study demonstrated the feasibility of implementing conventional CDT to be automatically evaluated by DNN with a fair performance in a larger scale than ever, suggesting its potential as a mass screening test for ruling-in or ruling-out those with executive dysfunction or with probable dementia.

## Introduction

The Clock-Drawing Test (CDT) is a cognitive test conventionally used for assessing multiple cognitive domains including executive function (1, 2). Because CDT is easy to use and can be conducted across different cultural backgrounds (2), it is sometimes used as a screening tool such as the Mini-Mental State Examination to identify individuals with cognitive decline. In Japan, CDT has been used as one of the screening tools in the drivers' license renewal process for individuals ≥ 75 years (which included > 1.5 million individuals in FY2017) to detect those who may have cognitive impairment that impedes their ability to drive cars (https://www.npa.go.jp/policies/application/license_renewal/ninti/index2.htm).

A potential barrier to implementing CDT for mass screening is its wide variability and the complexity of evaluating the drawn clock images (1). Assessment needs to be manually conducted by trained raters according to particular scoring criteria, which is time-consuming, and makes CDT essentially unsuitable to apply in a very large number of people at once. Therefore, if we could automatically evaluate CDT, the utility of CDT would increase considerably: potential uses include screening for eligible participants for clinical trials for Alzheimer's disease (3), and in clinical practice for outpatient visits (not limited to memory clinic).

A deep learning-based approach to automatically score the drawn clock images has been reported recently in some earlier studies that achieved 96% of accuracy in dementia screening (4) and 77% of accuracy in discriminating cognitively impaired individuals from cognitively normal individuals (5), thereby enabling automatic scoring of pictures to overcome the previously mentioned limitations of using CDT in screening. However, these earlier studies were conducted in a specific clinical setting (e.g., memory clinic), which means that there could be bias regarding the background of visiting patients, prior probability of being dementia, comorbid diseases, or the causes of cognitive decline. Therefore, the utility of CDT as a screening tool has not always been evaluated in a large cohort that is similar to the general population. As such, there is the need to simulate the situation whereby CDT will be implemented as a mass screening tool. In addition, the explainability of the model is one of the major concerns regarding the use of deep learning-based models (6) and would also need to be confirmed. To date, it remains unclear which aspects of the imputed CDT images were utilized for scoring in the DNN-based classifiers (5).

In this study, using sufficient CDT data obtained from a large cohort of older adults from the National Health and Aging Trends Study (NHATS), which collects annual interviews of nationally representative samples of older adults among Medicare beneficiaries in the United States (7), we aim to build a deep learning-based model to detect cognitive decline while confirming the explainability of the obtained models. Our attempt will not only contribute to obtain a CDT screening tool that is expected to be robust enough for use in mass screening, but also to ascertain the reproducibility of previous DNN-based CDT scoring models (4, 5).

## Methods

### Data From NHATS

This is a retrospective study using publicly distributed data from the NHATS (https://www.nhats.org). This study was approved by the University of Tokyo Graduate School of Medicine institutional ethics committee [ID: 11628-(3)]. Informed consent was not required because the study uses publicly distributed, anonymized data only. NHATS involves nationwide surveys conducted since 2011 comprising annual interviews with over 8,000 Medicare beneficiaries aged ≥ 65 years in the United States, to investigate late-life disability trends and trajectories (https://www.nhats.org) (7). A stratified random sampling of community-dwelling older adults is conducted to select the samples: the detailed study design and procedures are described in their previous technical reports (8). The data have been distributed annually, and we obtained data (round 1–9, which are collected in 2011–2019) from their website (https://nhats.org/researcher/data-access/public-use-files) in June 2021.

### NHATS Data Related to Cognitive Function

In NHATS, cognitive features including CDT images have been collected for every round from the participants (Supplementary Figure S1A): A number of eight questionnaires (i.e., today's day of week, day, month, year, and the name of the current president and vice president of the United States) to assess orientation (0–8 in score), 10-word recall questionnaires to evaluate memory (0–20 in score), and CDT to assess executive function (0–5 in score). The cutoff threshold to determine a significant decline in the study population is reported as mean−1.5 SD (8): namely, 0–3 in orientation or in recall, and 0–1 in CDT. These cognitive tests were conducted for all the participants for every round. Since all sample data of NHATS include the annual survey of the same individuals, we refer to individual survey results as “samples” and refer to unique individual participants as “cases”.

**Figure 1:**
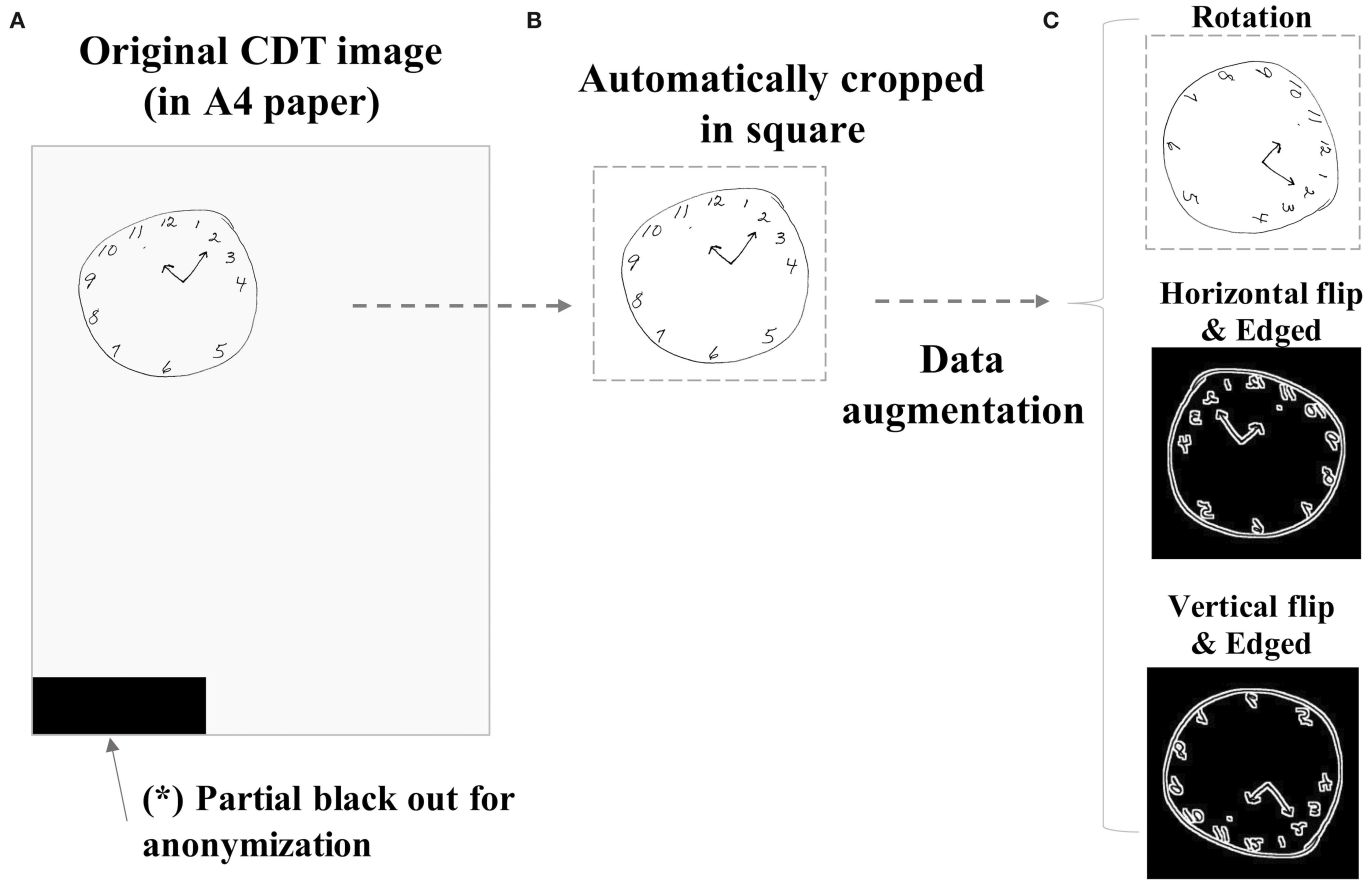
Sample image preprocessing procedure. The original CDT images drawn on A4-sized papers **(A)** were preprocessed by automatically cropping in a square **(B)**. The images were further augmented by several combinations of manipulations such as rotation (90 or 180°), horizontal flip, vertical flip, or black–white reversal by edging **(C)**. CDT, Clock-Drawing Test.

In the NHATS study, CDT images were obtained from each participant by having them draw a picture of analog clock with the hands showing 11:10 am on a blank A4 size paper (Figure 1A). The clock is to be drawn freely without a pre-defined outer frame, which is different from that employed in some previous studies analyzing CDT data (1, 4). Due to the dropout or exclusion from surveillance follow-up, the number of CDT images obtained from each case is not always equal. Every CDT image is scored by the interviewers according to the pre-defined criteria, using the range of 0–5 (Figure 3) as follows: (0) not recognizable as a clock, (1) severely distorted depiction, (2) moderately distorted depiction, (3) mildly distorted depiction, (4) reasonably accurate depiction, and (5) accurate depiction.

### Data Preprocessing

The following data handling and analyses are conducted using R statistical software (version 3.6.3, the R foundation) in macOS Catalina. To build a deep neural network (DNN) model to predict participants' cognitive status based on the drawn clock pictures, we used two different objective variables as targets to predict as follows:

1. With/without significant decline in executive function
2. With/without probable dementia

For (1), all CDT images were dichotomized (0/1), where CDT rating 0–1 corresponds to poorer performance (= 1 in binary) or 2–5 corresponds to better performance (= 0 in binary), as demonstrated in the earlier technical report (8). For (2), all CDT images were dichotomized (0/1) whether the case on the same round fulfills the criteria of probable dementia (8): the case exhibited abnormal performance in at least 2 of 3 cognitive categories [i.e., orientation (questionnaire score ≤ 3), memory (questionnaire score ≤ 3), and executive function (CDT score ≤ 1)].

**Figure 2:**
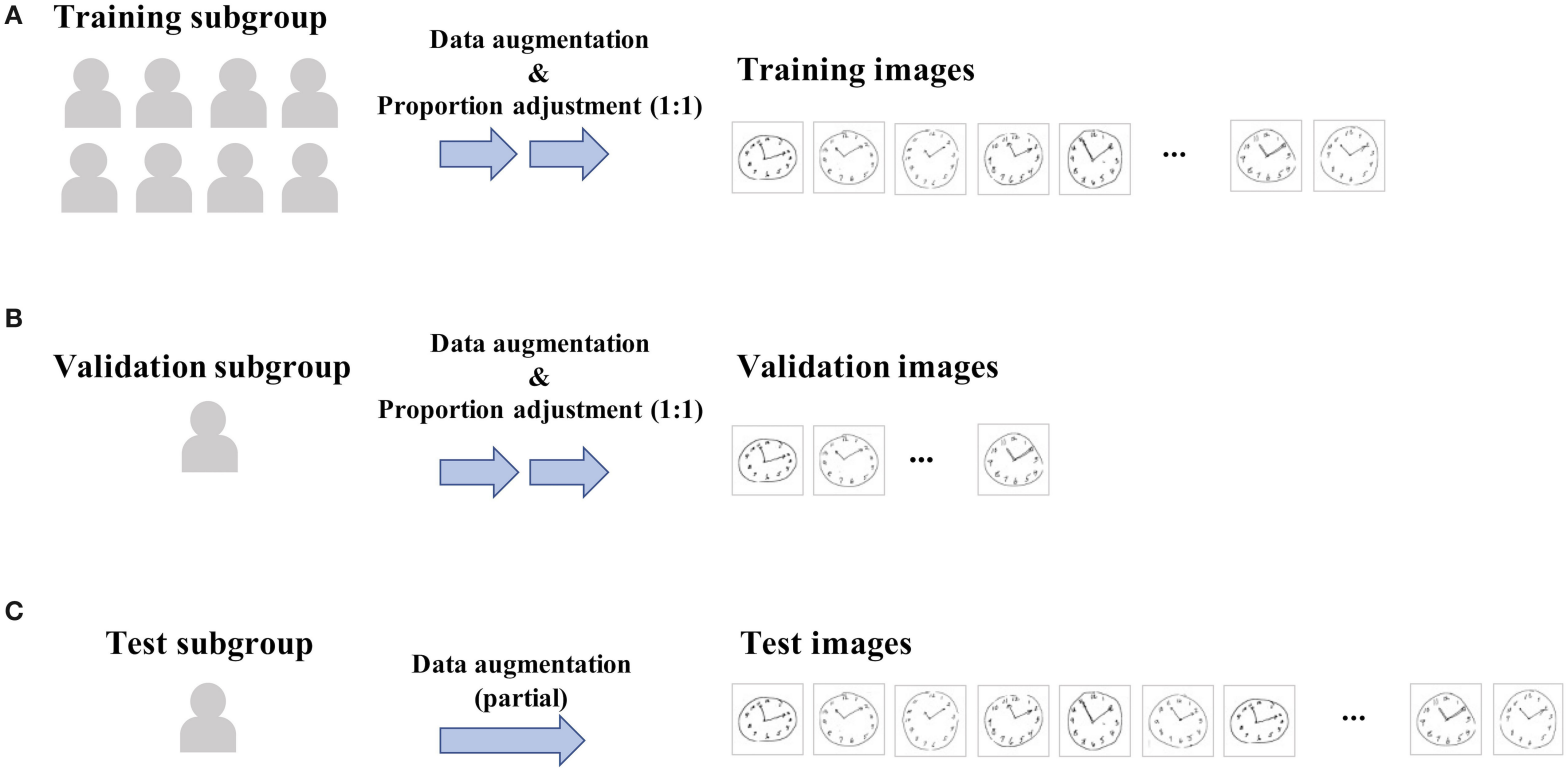
Data workflow for training and validating. All eligible cases were randomly split into three subgroups [i.e., training **(A)**, validation **(B)**, or test **(C)** subgroup] in an approximate 8:1:1 ratio, and then, the CDT image samples derived from these cases were to belong to the same subgroup as of cases. Data splitting was conducted so as to make the proportion of positive samples included in each subgroup being almost equal. Then, for training and validation subgroups, proportions in the positive vs. negative samples were further adjusted to be 1:1 within each subgroup by randomly downsampling negative samples, since the original sample data are highly imbalanced (e.g., proportion of cases with dementia is < 10%).

All eligible cases were randomly split into three subgroups (i.e., training, validation, or test) in an approximate 8:1:1 ratio (Figures 2A–C), and then, the CDT image samples derived from these cases were to belong to the same subgroup as of cases, regardless of the inter-participant variability in the dropout timing. This is because of the concern regarding data leakage between subgroups, since the same participant may draw such specific or similar clock pictures for every round that they can be identifiable by themselves. Data splitting was conducted by R package {*caret*}, enabling the target variables to be almost equally distributed between the subgroups.

Since the original CDT samples are by themselves not suitable to impute to DNN directly because of the small size of the clock drawn on paper, different directions of paper the CDT was drawn, or contamination of any other unrelated noises to blackout as in Figure 1A to mask identifiable information. Therefore, the raw images were preprocessed by automatically cropping in a square (Figure 1B) and resized to 112^*^112 regardless of the original image size. The images were further augmented to increase the number of samples to impute to DNN by adding the combination (Supplementary Table S1) of the following manipulations: rotation (90 or 180°), horizontal flip, vertical flip, or black–white reversal by edging (Figure 1C). For test subgroup samples, only original or rotated images were used to simulate actual settings.

Since the original sample data are highly imbalanced (e.g., the proportion of cases with dementia is <10%), proportions of positive vs. negative samples in training and validation subgroups were adjusted to be equal (i.e., 1:1) (Figures 2A,B). This was achieved by randomly downsampling negative samples from the training and validation subgroups, respectively. The same procedure was not conducted in the test subgroup.

### DNN Architecture

We applied the same layer architecture as that of “mini-VGG” (https://github.com/amrfodd/MINI-VGG-Architecture.git), although we have not used its ready-made network weights [e.g., transfer learning using Visual Geometry Group (VGG) network (9)]. This network comprises four sets of convolution and activation layers, thereafter fully-connected layers to discriminate 2 target classes (positive vs. negative) (Supplementary Figures S1B–D; name and output form (e.g., “32^*^32^*^32”) are shown in each layer). Kernel size was set to 3^*^3.

### Model Training and Validating

For training DNN classifier, we used R package {*keras*} (https://keras.rstudio.com). Training to increase the predictive accuracy and its validation were conducted in reference to the training and validation subgroup sample images, respectively, for a maximum epoch of 50 times. Categorical cross-entropy was used as a loss function (4). In minimizing or maximizing the loss function, *Adam* algorithm was used (4) with its default settings (e.g., learning rate = 0.001, decay = 0) in *keras*. The mini-batch size was set to 32. To terminate the learning process of training and validating, early stopping was arbitrarily applied based on the validation accuracy curve.

The obtained classifier was then applied to the test subgroup samples to eventually evaluate the performance of DNN models. Accuracy, sensitivity, and specificity, positive likelihood ratio (PLH), and negative likelihood ratio (NLH) were measured. Since the test subgroup comprises imbalanced data, we mainly refer to PLH and NLH but not accuracy to discuss the degree of performance of the obtained classifiers. We repeated trials using the same procedure–data split, training and validation, and test–for 10 times while setting different random seeds, and thereby obtained the averaged performance.

### Visualizing Extracted Features in DNN

Next, we apply gradient-weighted class activation mapping (Grad-CAM) (10) to visualize the features on which the DNN model focuses within CDT images to make a judgment. Alternative method such as layer-wise relevance propagation (11) was not used here because it was not available in the R keras toolchain. Output from the last convolutional layer as marked by a star in Supplementary Figure S1C was used for the analysis (https://github.com/rstudio/keras/issues/182). The obtained map was overdrawn on the original image, to assess the extent to which the DNN-based prediction may be valid visually. Heatmap coloring was made by R package {*viridis*}.

## Results

### Basic Features

We included 9,861 unique participants in total, among whom 57.1% (5,632/9,861) were women. The median age class was 75–79 years. We included 40,131 CDT images obtained from all participants. When focusing on the first-time sample for each participant, the proportion of cases with probable dementia was 1.54% (152/9,861), and the proportion of cases with executive dysfunction was 4.12% (406/9,861). The basic characteristics are provided in Table 1.

**Table 1:** Basic characteristics of participants.

**Objective variable**	**Target (****1****)** **(executive dysfunction or not)**		**Target (****2****)** **(probable dementia or not)**	
	**CDT ≤ 1**	**CDT ≥ 2**	**Probable dementia**	**others**
Total *N*	406	9,448	152	9,702
Median age (years)	80–84	75–59	80–84	75–79
Sex: female (%)	193 (47.5%)	5,439 (57.6%)	94 (61.8%)	5,538 (57.1%)

*CDT, Clock-Drawing Test*.

### Prediction Performance

**Figure 3:**
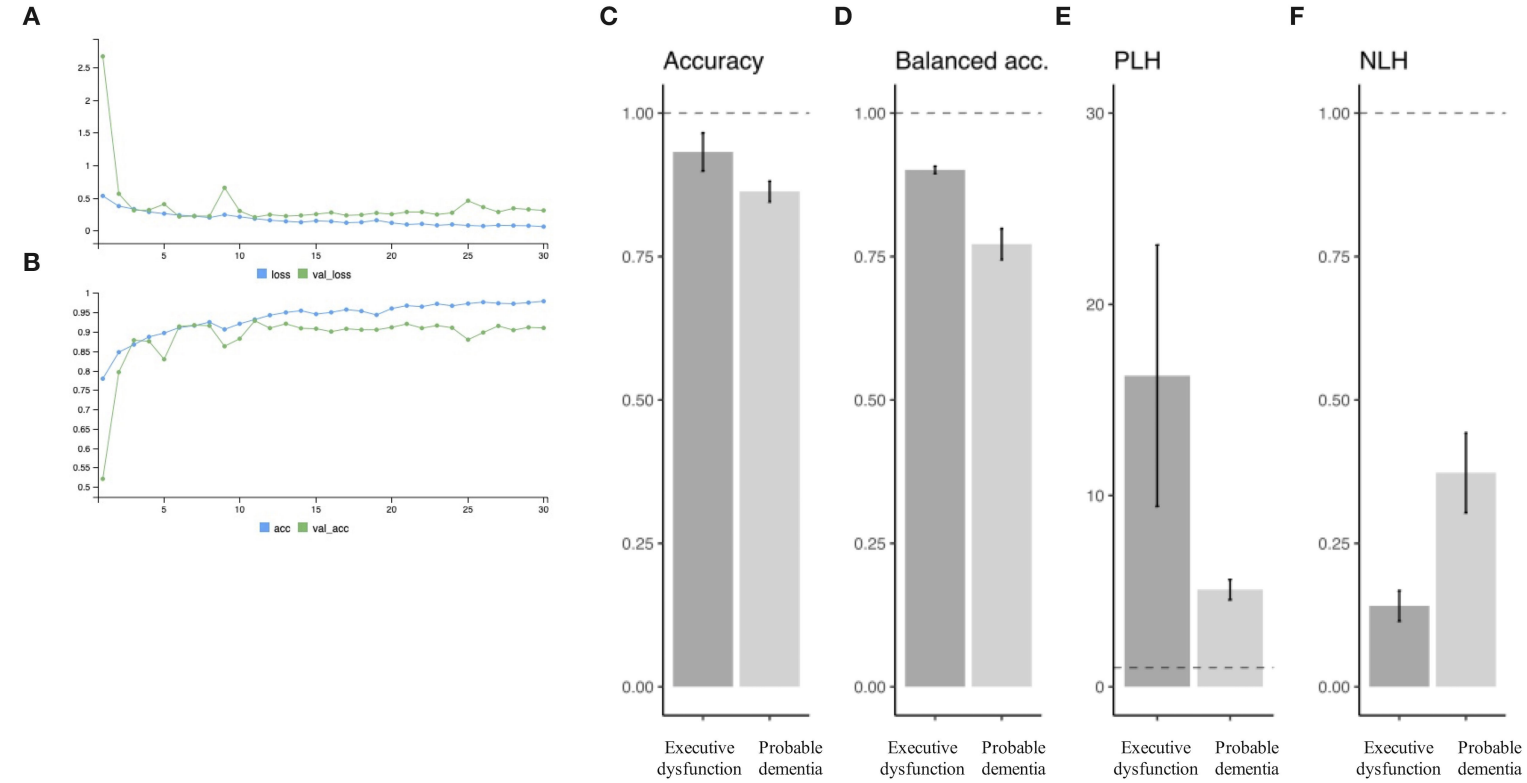
Prediction performance. An example of the learning curve for predicting executive dysfunction. Serial change is observed in loss function **(A)** and accuracy **(B)** as learning goes. Predictive performance is summarized in terms of accuracy **(C)**, balanced accuracy **(D)**, PLH **(E)**, and NLH **(F)** for each of the target variables (i.e., “executive dysfunction” or “probable dementia”). PLH, positive likelihood ratio; NLH, negative likelihood ratio.

Figures 3A,B show an example of the learning curve for predicting executive dysfunction: (Figure 3A) shows decreasing loss function in training and validation subgroups, and (Figure 3B) shows increasing accuracy in training and validation subgroups. In this curve, accuracy and loss function appeared to plateau around epoch = 15.

Overall performance in the test subgroup samples is summarized in Figures 3C–F in mean +/- SD by different target variables. For the target (1) (i.e., executive dysfunction or not), there were 93.2 +/- 3.3% of accuracy, 90.1 ± 0.6% of balanced accuracy, 16.3 +/- 6.8 of PLH, and 0.14 +/- 0.03 of NPH. Specificity was higher than sensitivity for all trials: for example, sensitivity = 0.719 and specificity = 0.879 when PLH = 5.95 (> 5) and NLH = 0.320 (> 0.20).

In addition, for the target (2) (i.e., probable dementia or not), there were 86.3 +/- 1.8% of accuracy, 77.2 ± 2.7% of balanced accuracy, 5.1 +/- 0.5 of PLH, and 0.37 +/- 0.07 of NPH. Specificity was also higher than sensitivity: for example, sensitivity = 0.847 and specificity = 0.953 when PLH = 18.1 (> 10) and NLH = 0.16 (<0.20).

### Class Activation Mapping

**Figure 4:**
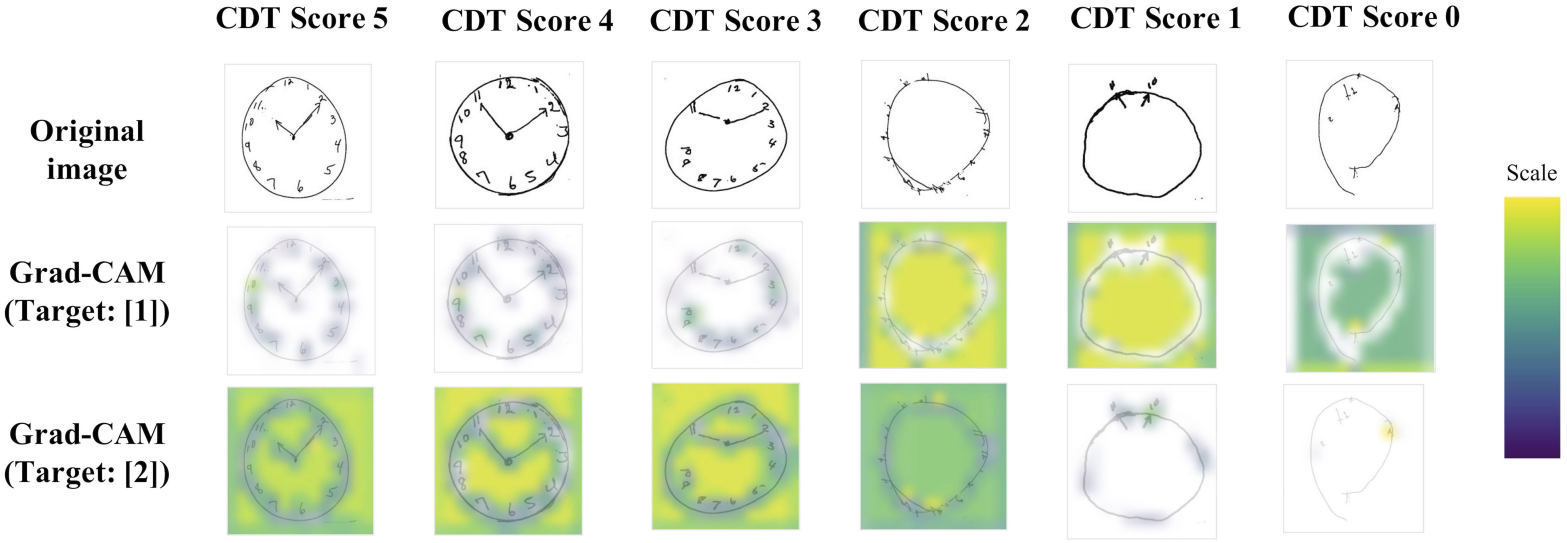
Grad-CAM examples. Examples of imputed CDT images and their extracted features through the CNN network. Yellow or green color corresponds to the higher weighted region of interest. For target (1), clock letters and hands were referred to within clock images with a better quality. Meanwhile, for target (2), not only clock letters, hands, and outer circle, but also clock background spaces were also referred to within clock images with a better quality. CDT, Clock-Drawing Test; Grad-CAM, gradient-weighted class activation mapping.

Next, we visualized Grad-CAM (10): Figure 4 shows the examples of imputed original images and their extracted features through the CNN network (Grad-CAM is overdrawn on the original images). Yellow or green color corresponds to the higher weighted region of interest. For target (1), clock letters and hands were referred to within clock images with a better quality. Meanwhile, for target (2), not only clock letters, hands, and outer circle, but also clock background spaces were also referred to within clock images with a better quality.

## Discussion

In this study, using CDT data obtained from NHATS, we created a DNN-based prediction model to detect cognitive decline. The achieved performance in balanced accuracy was ~90% for executive dysfunction and 77% for probable dementia, being similar to the performance in other DNN-based studies using CDT (4, 5). The unique characteristics of our study compared to these earlier studies are as follows: (1) we used sufficient CDT data obtained from a large cohort of older adults, which is much closer to that of the general population than that of the memory clinic, and (2) we visually confirmed some extent of validity of feature extraction of the DNN models by class activation mapping.

These features suggest the potential of CDT as a mass screening tool for cognitive decline in the general older population, of which clinical settings are raised as follows: CDT may become more easily utilized in the outpatient clinic for primary care but not limited to specialized care (e.g., memory clinic). CDT can be included as one of the contents in routine health checkup or can also be conducted in long-term care facilities, to identify elderly individuals with unknowingly deteriorating cognition. In these contexts, PLH for predicting those with probable dementia was 5.1 (> 5.0), suggesting that the CDT possesses some degrees of reliability in identifying participants whose cognitive level is mild cognitive impairment or dementia.

In addition, CDT may also be utilized in the screening process of Alzheimer's disease (AD) prevention trials, of which the background is described as follows. Recent advances in the development of AD prevention drugs highlight the importance of enrolling individuals with no or limited cognitive impairment (12, 13) (e.g., asymptomatic “preclinical AD” or mild cognitive impairment). Because the target individuals are likely to have mild symptoms, there are some attempts to recruit participants *via* web-based registry (3). In this context, since individuals whose cognitive decline had progressed substantially are not eligible for such AD prevention trials, we wondered whether it is possible that the CDT can be incorporated into the online registries to help to exclude those with apparent executive dysfunction or the possibility of probable dementia, thereby facilitating an increase in the probability of participants being eligible for AD prevention trials. In the context of such mass screening to facilitate AD prevention trials, we should focus on NLH to rule out those who may have dementia: NLH in the prediction for those with probable dementia was 0.37, suggesting that CDT is not always a reliable screening tool to rule out non-eligible participants. NLH for those with executive dysfunction was 0.14 (< 0.20), so that DNN-based automated CDT exhibits some degrees of reliability as a screening test for executive function.

Based on the PLH and NLH, the performance of models was generally better in predicting those with executive dysfunction than in predicting those with probable dementia, which can be explained not only by the difference in the number of positive samples but also in terms of the cognitive domain corresponding to CDT (e.g., executive dysfunction). AD as the most prevalent cause of dementia (14) is known to frequently cause memory impairment during its early phase compared to other cognitive domain disabilities; therefore, it is understandable that the performance of CDT-based prediction for probable dementia was poorer than that for executive dysfunction.

The DNN architecture and hyper-parameter tuning used in this study may not have been the best, and there remains room to improve the prediction performance to a state-of-art level. Using transfer learning by fine-tuning a ready-made image classifier should also be considered (15). Achieving the highest performance was not our primary objective; however, even at such a level of parameter tuning, we could confirm that the prediction performance was similar to those of earlier reports. This confirms the reliability and robustness of CDT as a screening tool regardless of the settings in which the CDT is obtained.

Another approach to achieve good prediction accuracy would be first to extract and quantify all parameters of a drawn clock through image processing, such as the center dot of a clock, lengths of horizontal and vertical axes of clock, number, angle, and length of clock hands, or number and location of clock digits (16). Then, making machine-learning models to predict CDT scoring or the diagnosis of dementia based on these parameters was done in an earlier open challenge (https://www.aicrowd.com/challenges/addi-alzheimers-detection-challenge). Although this approach is not as straightforward as our direct-imputing approach is, it would still have an advantage regarding some additional points that its extracted features can also be referred to in the known scoring systems (1).

A digital device to measure CDT performance has been developed (17–19), which measures hand movements during the drawing of the clock image and to predict cognitive status (18). It should have far greater potential than that of conventional CDT in terms of its ability to obtain abundant mechanical information during drawing. However, this study using samples with conventional CDT would still possess some advantages, such as in the availability of considerable accumulated past data, or greater accessibility than the hand-tracking digital pencil has. It requires a specific testing device, which is not convenient for use by a large number of web-based study participants (20).

Our study has some limitations. First, CDT scoring in the NHATS does not provide detailed criteria so that there is a risk that depends on the interviewers' subjective evaluation. This can cause inconsistency in the scoring across all samples, leading to a limitation in the improvement of prediction accuracy. This is one of the reasons why we had not used the CDT score itself as a target to predict. One possible solution for this issue is to re-score all the clock images manually, so that we can obtain classifiers that are applicable to multiple types of CDT scoring systems.

Second, the quality of automatic cropping in the preprocessing (Figure 1) may not always be optimized, and some clock images with too poor quality (e.g., a mere circle with distorted shape, or a small circle) could have been removed from the analysis during the preprocess procedure. This would account for a slightly smaller proportion of probable dementia or executive dysfunction than those actually reported in the earlier technical paper by the original NHATS study team (8).

Third, although we considered that population background and the samples used in our study might have simulated much closer settings and discussed a hypothetical situation where a web-based approach was to be employed in the actual AD prevention trials, the actual applicability of CDT to web-based application has not been validated and needs to be verified in future studies. Remote CDT is also different from conventional CDT in terms of the easiness to cheat (e.g., watching clock in their room during the test). Although tablet-based or mobile phone-based CDT would be technically feasible as reported in an earlier study (20), it might be more difficult for patients with dementia to complete digital CDT on the tablet than in completing conventional CDT, so that we must expect a higher dropout rate when using CDT in remote online screening.

Fourth, we could not include participant basic features such as age, sex, education history, or family history, which are important features in predicting dementia, because this study was a CNN-based approach. Machine-learning approach inputting these features in combination with the CDT-based probability for being dementia may be helpful to further increase the prediction accuracy. In addition, we also could not exclude potential cases with specific medical history such as semi-lateral visual neglect which might interfere with the appropriate CDT testing. This is because it was impossible to detect such participants from the database.

To conclude, the current DNN-based study using CDT achieved similar performance as of earlier studies in terms of accuracy but in a larger scale than ever, suggesting the feasibility of implementing the conventional CDT as one of the mass screening tools for detecting decreased executive function or the status of probable dementia, thereby enhancing clinical practice and facilitating clinical studies for dementia in the post-pandemic era.

## Data Availability Statement

Publicly available datasets were analyzed in this study. This data can be found at: National Health and Aging Trends Study (NHATS), https://www.nhats.org.

## Ethics Statement

The study was approved by The University of Tokyo Graduate School of Medicine institutional Ethics Committee [ID: 11628-(3)]. Written informed consent for participation was not required for this study in accordance with the national legislation and the institutional requirements.

## Author Contributions

KS: conceptualization, data curation, analysis, and drafting manuscript. YN, TM, and AI: reviewing the manuscript. TI: reviewing the manuscript and supervision. All authors contributed to the article and approved the submitted version.

## Funding

This study was supported by JSPS KAKENHI Grant Number JP21K20891 and AMED Grant Number JP22dk0207048.

## Conflict of Interest

The authors declare that the research was conducted in the absence of any commercial or financial relationships that could be construed as a potential conflict of interest.

## Publisher's Note

All claims expressed in this article are solely those of the authors and do not necessarily represent those of their affiliated organizations, or those of the publisher, the editors and the reviewers. Any product that may be evaluated in this article, or claim that may be made by its manufacturer, is not guaranteed or endorsed by the publisher.

## Supplementary Material

Supplementary Figure S1 Data constitution and DNN architecture. Cognitive features including CDT images have been collected in NHATS study for every round from the participants **(A)**. Due to the dropout or exclusion from surveillance follow-up, the number of CDT images obtained from each case is not always equal. For DNN network, we used the same layer architecture as that of “mini-VGG.” This network comprises 4 sets of convolution and activation layers **(B,C)**, thereafter fully-connected layers to discriminate 2 target classes (positive vs. negative) **(D)**. DNN, deep neural network; ReLU, rectified linear unit.

Supplementary Table 1 Combination of manipulations for data augmentation. For the test subgroup, only not-edged rotation (cells with gray color) was applied for data augmentation.
