# Supplementary figures and images for: Automated Evaluation of Conventional Clock-Drawing Test Using Deep Neural Network: Potential as a Mass Screening Tool to Detect Individuals With Cognitive Decline
Source: Front Neurol. 2022 May 3;13:896403. doi: 10.3389/fneur.2022.896403 (PMC9110693; doi:10.3389/fneur.2022.896403)

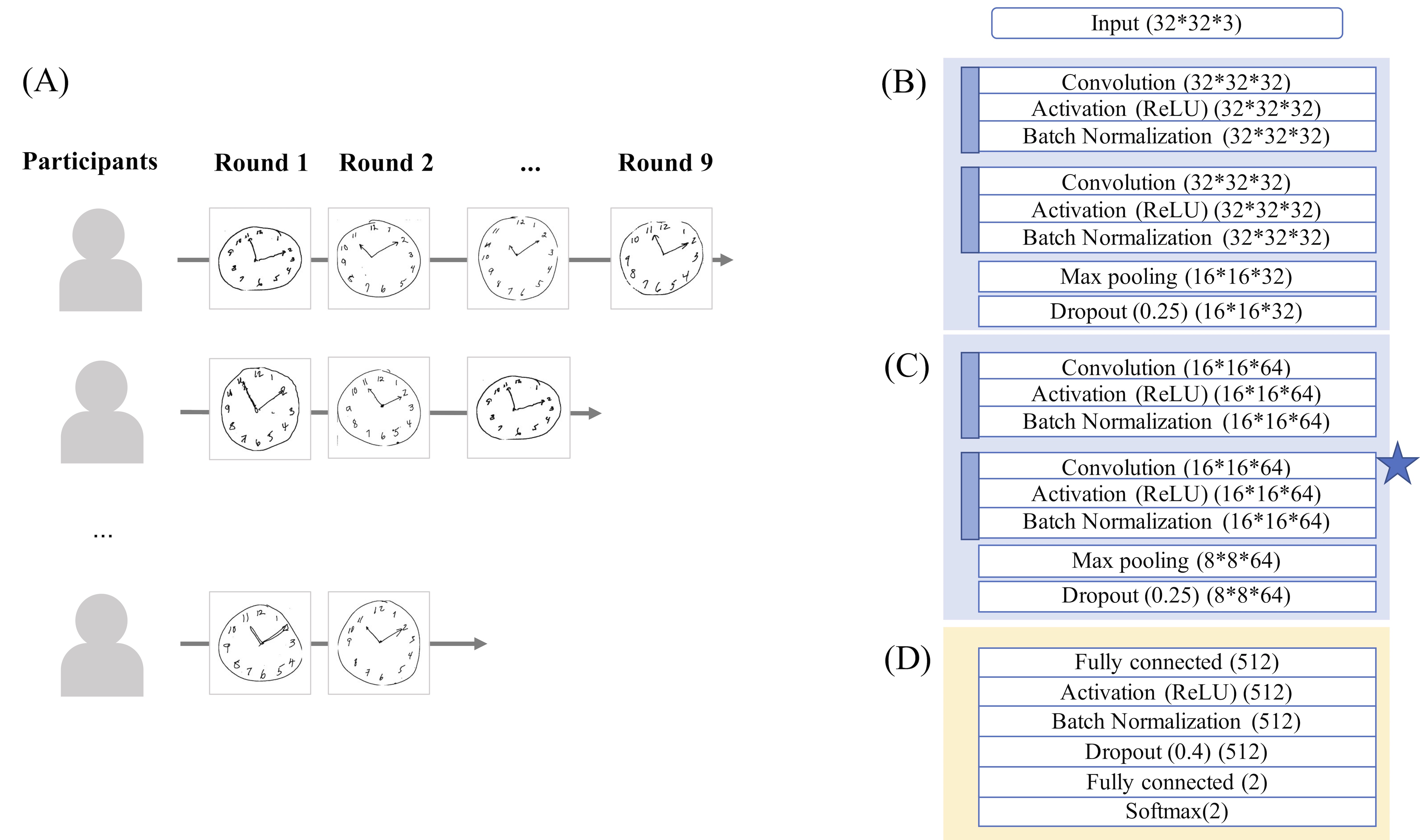

Supplement: Supplementary Figure S1 — Data constitution and DNN architecture. Cognitive features including CDT images have been collected in NHATS study for every round from the participants (A). Due to the dropout or exclusion from surveillance follow-up, the number of CDT images obtained from each case is not always equal. For DNN network, we used the same layer architecture as that of “mini-VGG.” This network comprises 4 sets of convolution and activation layers (B,C), thereafter fully-connected layers to discriminate 2 target classes (positive vs. negative) (D). DNN, deep neural network; ReLU, rectified linear unit. [file Image_1.JPEG]
