# Supplementary material for: Automated Evaluation of Conventional Clock-Drawing Test Using Deep Neural Network: Potential as a Mass Screening Tool to Detect Individuals With Cognitive Decline
Source: Front Neurol. 2022 May 3;13:896403. doi: 10.3389/fneur.2022.896403 (PMC9110693; doi:10.3389/fneur.2022.896403)
Supplement: Supplementary Table 1 — Combination of manipulations for data augmentation. For the test subgroup, only not-edged rotation (cells with gray color) was applied for data augmentation. [file Table_1.docx]

Supplemental Table 1. Combination of manipulations for data augmentation

|  | Rotation | | | Horizontal flip | Vertical flip |
| --- | --- | --- | --- | --- | --- |
|  | 0 degree | 90 degree | 180 degree |  |  |
| Not edged | (original) |  |  |  |  |
| Edged |  |  |  |  |  |

For the test subgroup, only not-edged rotation (cells with gray color) was applied for data augmentation
